# The effects of probiotic supplementation on lean body mass, strength, and power, and health indicators in resistance trained males: a pilot study

**DOI:** 10.1186/1550-2783-11-S1-P38

**Published:** 2014-12-01

**Authors:** John Georges, Ryan P Lowery, George Yaman, Chris Kerio, Jacob Ormes, Sean A McCleary, Matthew Sharp, Kevin Shields, Jacob Rauch, Jeremy Silva, Ned Arick, Martin Purpura, Ralf Jäger, Jacob M Wilson

**Affiliations:** 1 The University of Tampa, Tampa, Florida, USA; 2 Increnovo LLC, Milwaukee, Wisconsin, USA

## Background

While growing evidence suggests beneficial effects of probiotics on the gut-brain-axis, only a limited number of studies have investigated the impact of gut microbiota modulation on muscle physiology (gut-muscle-axis). The probiotic BC30 (Ganeden Biotech Inc., Maryfield Heights, OH) has been shown to increase protein absorption and the anabolic potential of a respective protein source has been directly linked to peak plasma leucine levels. Post-workout administration of slow digesting proteins such as casein show inferior results on muscle protein synthesis in comparison to fast absorbed proteins such as whey. Thus, the purpose of this investigation was to determine if the co-administration of a probiotic with a slow digested protein has a beneficial effect on body composition, performance, and measures of perceived health.

## Methods

10 healthy resistance-trained individuals volunteered to participate in this study (mean+/-SD; age: 22.0 ± 2.4 yr; height: 181.8 ± 4.1 cm; weight: 85.6 ± 12.9 kg). Subjects were randomly assigned to consume either 20g of casein (Control = CON) or 20g of casein plus probiotic (500M BC30, =BC30) twice daily. Subjects were instructed to consume one serving in the morning upon waking while the second serving was consumed after training or before bed on non-training days. With assistance from a dietician, macronutrients were controlled to 50% carbohydrate, 25% protein, and 25% fat between groups using the Mifflin-St Jeor formula. Subjects performed full body workouts 4-times per week for 8 weeks consisting of hypertrophy (8-12 RM loads and 60 seconds rest), and strength (1-5 RM loads with 3-5 minutes rest) under supervision of the researchers in order to ensure compliance. Body composition (Dual X-Ray Absorptiometry; DXA), quadriceps thickness (ultrasound), peak power (Monark Wingate Cycle), vertical jump power (Tendo unit), 1-RM bench press, and 1-RM leg press were measured at baseline and after the eighth week of supplementation. Perceived GI health (GSRS) was measured weekly and upper respiratory health (WURSS-21) daily. Consent to publish the results was obtained from all participants.

## Results

BC30 showed a trend (p=0.10) to increase vertical jump power (BC30: pre 2,136 W, post 2,262 W; CON pre 1,712 W, post 1,691 W) and might have a beneficial effect on peak power and fat mass. There were no significant differences between groups for body composition, or other performance measures. Due to an overall very low number of incidences in digestive and immune health in both groups no meaningful analysis could be done.

## Conclusions

This pilot study indicated that probiotic supplementation in form of BC30 in combination with a slow digesting protein might increase athletic performance. However, further research with a larger n-size is needed to confirm these findings.

